# Effectiveness of Second mRNA COVID-19 Booster Vaccine in Immunocompromised Persons and Long-Term Care Facility Residents

**DOI:** 10.3201/eid2811.220918

**Published:** 2022-11

**Authors:** Yoo-Yeon Kim, Young June Choe, Jia Kim, Ryu Kyung Kim, Eun Jung Jang, Seon Kyeong Park, Do-Sang Lim, Seonju Yi, Sangwon Lee, Geun-Yong Kwon, Jee Yeon Shin, Sang-Yoon Choi, Mi Jin Jeong, Young-Joon Park

**Affiliations:** Korea Disease Control and Prevention Agency, Cheongju, South Korea (Y.-Y. Kim, J. Kim, R.K. Kim, E.J. Jang, S.K. Park, D.-S. Lim, S. Yi, S. Lee, G.-Y. Kwon, J.Y. Shin, S.-Y. Choi, M.J. Jeong, Y.-J. Park);; Korea University Anam Hospital, Seoul, South Korea (Y.J. Choe)

**Keywords:** COVID-19, coronavirus disease, SARS-CoV-2, severe acute respiratory syndrome coronavirus 2, viruses, respiratory infections, zoonoses, vaccine, booster, mRNA, South Korea

## Abstract

We used a nationwide population registry in South Korea to estimate the effect of a second booster dose of mRNA COVID-19 vaccine on the risk for laboratory-confirmed SARS-CoV-2 infection, critical infection, and death in immunocompromised persons and long-term care facility (LTCF) residents. During February 16–May 7, 2022, among 972,449 eligible persons, 736,439 (75.7%) received a first booster and 236,010 (24.3%) persons received a second booster. Compared with the first booster group, at 30–53 days, the second booster recipients had vaccine effectiveness (VE) against all infections of 22.28% (95% CI 19.35%–25.11%), VE against critical infection of 56.95% (95% CI 29.99%–73.53%), and VE against death of 62.96% (95% CI 34.18%–79.15%). Our findings provide real-world evidence that a second booster dose of mRNA vaccine substantially increases protection against critical infection and death in these high-risk population groups.

Booster doses of mRNA vaccines have been shown to reduce the risk for severe SARS-CoV-2 infection (*1*,*2*); however, protection wanes a few months after vaccination, particularly in high-risk populations (*3*,*4*). A second booster dose at least 4 months after the first booster dose of mRNA vaccine was found to increase immunity against COVID-19; thus, the second booster has been introduced in some countries (*5*).

Other studies have postulated that additional doses of the COVID-19 vaccine can enhance cellular and humoral immunity against the Omicron variant (*6*,*7*), and some studies have already shown the effectiveness of second boosters in preventing COVID-19 infections (*5*,*8*,*9*). However, population-based studies are needed to assess the effect of the second booster vaccine on COVID-19 in high-risk groups.

During February–April 2022, a second booster of mRNA vaccine was recommended for immunocompromised persons and long-term care facility (LTCF) residents in South Korea. We used a nationwide population registry to estimate the effect of a second booster dose of mRNA vaccine on risk for laboratory-confirmed SARS-CoV-2 infection, critical infection, and death in immunocompromised persons and LTCF residents.

## Methods

In South Korea, COVID-19 is a notifiable disease; all laboratory-confirmed cases are reported to the Korea Disease Control and Prevention Agency (KDCA). COVID-19 vaccination records, including the date of vaccination and type of vaccine, are also collected and maintained by the KDCA. All suspected COVID-19 case-patients (anyone with a history of close contact with a COVID-19 patient) or SARS-CoV-2–infected persons, regardless of symptoms, were mandated to be tested by PCR or rapid antigen test during the observation period. By linking the vaccination registry and the surveillance database, we created a large-linked database through unique resident registration number. The observation period was February–May 2022, when 100% of SARS-CoV-2 detected in South Korea was identified as an Omicron variant (BA1.1, BA2, and BA2.3 subvariants) (*10*).

In South Korea, 2 adenoviral vector-based vaccines, ChAdOx1 nCov-19 (AstraZeneca, https://www.astrazeneca.com) and Ad26.COV2.S (Johnson & Johnson/Janssen, https://www.jng.com); 2 mRNA-based vaccines, BNT162b2 (Pfizer-BioNTech, https://www.pfizer.com) and mRNA-1273 (Moderna, https://www.moderna.com); and 1 protein subunit vaccine (Novavax, https://www.novavax.com) were introduced. Since February 2021, all immunocompromised persons and LTCF residents have been prioritized to receive COVID-19 vaccines (*11*). The first booster dose of Pfizer-BioNTech or Moderna vaccine has been offered since October 2021, and the second booster dose of Pfizer-BioNTech or Moderna vaccine has been offered since February 2022. We included all immunocompromised persons and LTCF residents who received the first booster vaccine >120 days before the study.

We defined immunocompromised persons as cancer patients, transplant patients, patients with primary immune deficiencies, patients with human immunodeficiency virus infections, and patients receiving high-dose corticosteroids or immunosuppressants. We defined having the first booster vaccine as the third dose of vaccination after receiving 2 doses of the primary series of AstraZeneca vaccine, Pfizer-BioNTech vaccine, or Moderna vaccine and second booster dose as reaching day 14 after receiving the fourth dose of vaccine.

We examined the 3 health outcomes of infection, critical infection, and death during the period of day 0–53. We defined day 0 as the 14th day after receiving the second booster dose. We defined infection as a SARS-CoV-2–positive PCR or rapid antigen test conducted by a healthcare professional in any symptomatic or asymptomatic patient and critical infection as illness in hospitalized SARS-CoV-2–positive patients that necessitated high-flow oxygen therapy, mechanical ventilation, extracorporeal membrane oxygenation, or continuous renal replacement therapy or that resulted in death within 28 days after laboratory confirmation of SARS-CoV-2. Death was death attributable to COVID-19 as diagnosed by physicians.

We compared the rates of all infections, critical infections, and deaths by sex, age, geographic region, and number of vaccinations in immunocompromised persons and LTCF residents. We computed the cumulative incidence curves of all infections, critical infections, and deaths in the first booster group versus second booster group using the Kaplan–Meier estimator. We used a time-dependent Cox proportional hazard model and estimated hazard ratios (HRs) with 95% CIs from an adjusted Cox model with covariates (sex, age, days elapsed since vaccination, census regions, residence in a facility, and immunocompromised status) to compare the rates. We calculated vaccine effectiveness (VE) for the second booster compared with the first booster in preventing infection, critical infection, and death by using the HR from this model: vaccine effectiveness (against all infections, critical infections, and death) = (1 – HR) × 100. We calculated time-varying VE 0–14 days, 15–30 days, and >30 days after the second booster vaccine. We used R software (The R Project for Statistical Computing, https://www.r-project.org) to prepare the data and perform statistical analyses.

This study was conducted as a legally mandated public health investigation under the authority of the Korean Infectious Diseases Control and Prevention Act (Nos. 12,444 and 13,392) and was not subject to institutional review board approval; therefore, written informed consent was not required. The investigators shared anonymized clustered data. 

## Results

**Figure 1:**
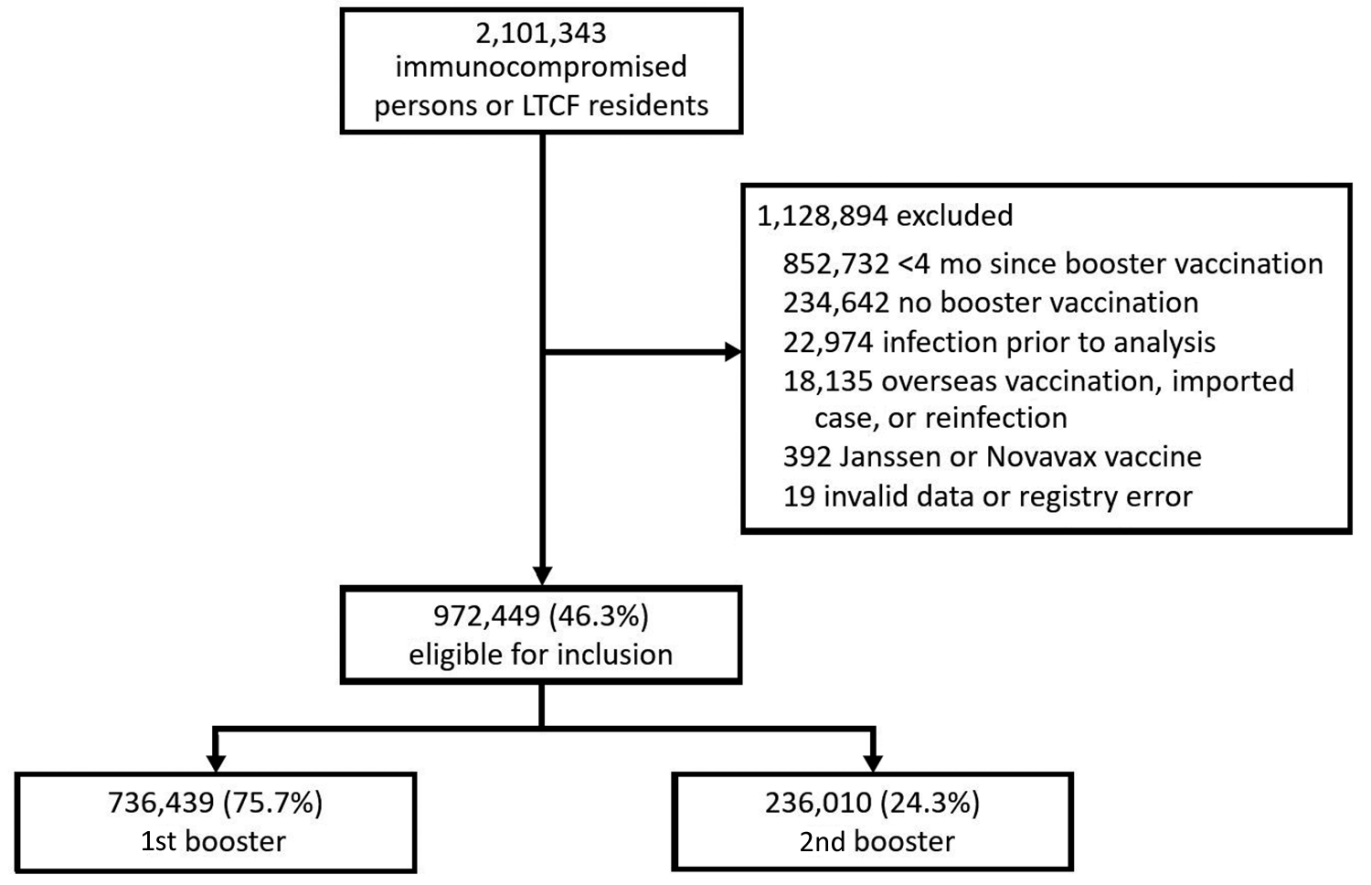
Flowchart of COVID-19 vaccine effectiveness study among immunocompromised persons and LTCF residents, South Korea, February–May 2022. Johnson & Johnson/Janssen, https://www.jng.com; Novavax, https://www.novavax.com. LTCF, long-term care facility.

During February 16–May 7, 2022, a total of 2,101,343 persons were assessed for inclusion, of whom 972,449 (46.3%) were eligible (Figure 1). A total of 736,439 (75.7%) of these persons had received the first booster dose, and 236,010 (24.3%) received the second booster dose.

The observed periods were 39,175,439 person-days for the first booster group and 5,197,160 person-days for the second booster group (Table 1). Immunocompromised patients accounted for 64.8% (n = 477,215) of the first booster group and 41.3% (n = 97,478) of the second booster group.

**Table 1:** Characteristics among immunocompromised persons and LTCF residents in COVID-19 vaccine effectiveness study, South Korea, February–May 2022

Characteristics	1st booster				2nd booster		
No. (%) participants		Person-days	No. (%) participants		Person-days
Total	736,439		39,175,439		236,010		5,197,160
Sex							
F	294,572 (40.0)		16,096,170		94,864 (40.2)		2,129,509
M	441,867 (60.0)		23,079,269		141,146 (59.8)		3,067,651
Age group, y							
18–39	33,010 (4.5)		1,609,745		8,811 (3.7)		196,656
40–59	171,954 (23.3)		8,916,315		57,425 (24.3)		1,233,939
60–74	332,987 (45.2)		17,958,470		96,829 (41.0)		2,165,276
>75	198,488 (27.0)		10,690,910		72,945 (30.9)		1,601,290
Location							
Metropolitan area	344,722 (46.8)		18,053,072		95,738 (40.6)		2,084,863
Nonmetropolitan area	391,717 (53.2)		21,122,367		140,272 (59.4)		3,112,297
Risk factors							
Immunocompromised	477,215 (64.8)		25,564,786		97,478 (41.3)		2,132,538
Long-term care facility residents	259,224 (35.2)		13,610,653		138,532 (58.7)		3,064,622

During the 44,372,598 person-days of follow-up, 313,388 infections, 2,951 critical infections, and 2,441 deaths occurred (Table 2). Of all infections, 85.6% (n = 268,278) occurred in the first booster group, and 88.5% (n = 2,148) of deaths occurred in the first booster group. Of all infections, 37.6% (n = 117,935) were in persons 60–74 years of age, whereas 79.3% (n = 2,339) of critical infections and 83.7% (n = 2,043) of deaths were in persons >75 years of age.

**Table 2:** Result of booster vaccination among immunocompromised persons and LTCF residents in COVID-19 vaccine effectiveness study, South Korea, February–May 2022*

Characteristics	Population			All infection, no. (%)		Critical infection, no. (%)		Death, no. (%)	
	No. (%)		Person-days						
Total	972,449		44,372,598	313,388		2,951		2,441	
1st booster	736,439 (75.7)		39,175,439	268,278 (85.6)		2,609 (88.4)		2,148 (88.0)	
2nd booster	236,010 (24.3)		5,197,160	45,110 (14.4)		342 (11.6)		293 (12.0)	
Sex									
F	583,013 (60.0)		26,146,920	206,478 (65.9)		1,236 (41.9)		1,474 (60.4)	
M	389,436 (40.0)		18,225,678	106,910 (34.1)		1,715 (58.1)		967 (39.6)	
Age group, y									
18–39	41,821 (4.3)		1,806,401	17,391 (5.5)		4 (0.1)		1 (0.0)	
40–59	229,379 (23.6)		10,150,253	86,230 (27.5)		97 (3.3)		62 (2.5)	
60–74	429,816 (44.2)		20,123,745	117,935 (37.6)		511 (17.3)		335 (13.7)	
>75	271,433 (27.9)		12,292,199	91,832 (29.3)		2,339 (79.3)		2,043 (83.7)	
Location									
Metropolitan area	440,460 (45.3)		20,137,934	137,973 (44.0)		1,328 (45.0)		1,012 (41.5)	
Nonmetropolitan area	531,989 (54.7)		24,234,664	175,415 (56.0)		1,623 (55.0)		1,429 (58.5)	
Risk factors									
Immunocompromised	574,693 (59.1)		27,697,324	124,950 (39.9)		531 (18.0)		339 (13.9)	
LTCF residents	397,756 (40.9)		16,675,274	188,438 (60.1)		2,420 (82.0)		2,102 (86.1)	

*LTCF, long-term care facility.

**Figure 2:**
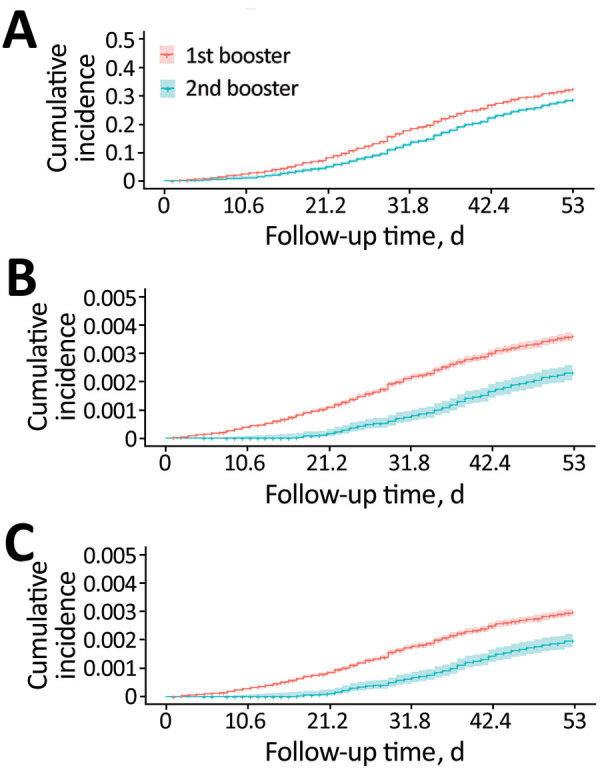
Cumulative incidence of all infections (A), critical infections (B), and death (C) in persons who received a second COVID-19 booster vaccination compared with those who received only the first booster dose in study of vaccine effectiveness among immunocompromised persons and long-term care facility residents, South Korea, February–May 2022.

**Figure 3:**
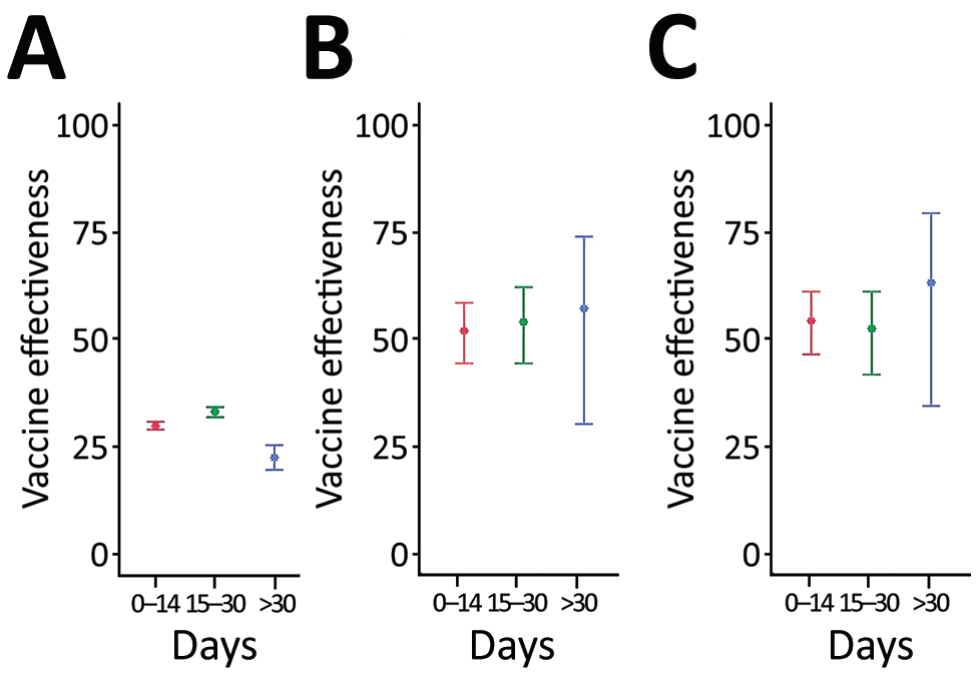
Time-varying COVID-19 vaccine effectiveness against all infections (A), critical infections (B), and death (C) in persons who received a second booster vaccination compared with those who received only the first booster dose in study of vaccine effectiveness among immunocompromised persons and long-term care facility residents, South Korea, February–May 2022. Error bars indicate 95% CIs.

We calculated time-varying VE against all infections, critical infections, and deaths in persons who received the second booster vaccination (Table 3; Figures 2, 3). At >30 days after the second booster vaccination, VE against all infections was low at 22.28% (95% CI 19.35%–25.11%), whereas VE was higher against both critical infection at 56.95% (95% CI 29.99%–73.53%) and against death at 62.96% (95% CI 34.18%–79.15%) (Figure 3).

**Table 3:** Incidence of SARS-CoV-2 infection, critical infection, and death among immunocompromised persons and LTCF residents after first and second mRNA booster vaccine, South Korea, February–May 2022

Category	Follow-up time, d					
0	10.6	21.2	31.8	42.4	53.0
All infections						
First booster						
No. at risk	972,449	937,931	822,346	653,579	540,367	470,791
No. events	0	24,129	76,592	158,059	227,409	268,278
Second booster						
No. at risk	850	10,365	72,142	150,436	175,606	192,093
No. events	0	24	1,369	10,375	29,070	45,110
Critical infections						
First booster						
No. at risk	972,449	937,931	822,346	653,579	540,367	470,791
No. events	0	377	998	1,762	2,298	2,609
Second booster						
No. at risk	850	10,365	72,142	150,436	175,606	192,093
No. events	0	0	8	68	218	342
Deaths						
First booster						
No. at risk	972,449	937,931	822,346	653,579	540,367	470,791
No. events	0	283	782	1,445	1,901	2,148
Second booster						
No. at risk	850	10,365	72,142	150,436	175,606	192,093
No. events	0	0	5	58	193	293

## Discussion

In this study that included ≈970,000 persons at high risk, we found that the second booster of mRNA vaccines provided greater protection against critical infection and death in patients with the Omicron variant than the first booster vaccination alone. Our finding is consistent with previous results, including a study among LTCF residents in Sweden during a period of Omicron variant predominance in which the effectiveness of the second booster dose compared with the first booster dose alone was reported to range from 31% to 42% against all-cause death (*12*). Our relative VE estimates were slightly lower than those in a study from Israel (HR 0.22–0.36), which might reflect declines in VE because the population in our study was a high-risk group consisting of immunocompromised persons and LTCF residents (*8*). In the general population >60 years of age in Israel, the estimated VE after the second booster dose was 45% against laboratory-confirmed infection, 55% against symptomatic infection, and 75% against death (*9*). A systematic review found that the seroconversion rates after COVID-19 vaccination were lower in immunocompromised patients, which might explain the difference in VE between populations (*13*). Despite this factor, our findings indicate that a second booster dose lowered the risk for severe infection in LTCF residents and immunocompromised persons, the most vulnerable population in the community. On the basis of these results, we recommend a second booster dose in at-risk populations to maximize the public benefit of protection against COVID-19 related illness and death.

Our findings also suggest that the second booster dose offers higher levels of protection against critical infection and death in immunocompromised persons and LTCF residents, who are at highest risk for severe COVID-19. A systematic review of 11 studies showed that a third dose of the mRNA vaccine was associated with seroconversion among vaccine nonresponders with malignancies and immune-mediated disorders, which is consistent with our findings (*13*). The immunogenicity in immunocompromised persons might be lower than that in immunocompetent persons; however, the second booster dose clearly provides additional protection in this population (*14*). The relatively low VE against all infections seems to be consistent with previous studies that examined VE in LTCFs; however, its effectiveness against severe infection or death was relatively sustained, as observed elsewhere (*15*,*16*). During the observation period, we saw no clear evidence of waning against critical infection or death >30 days after the second booster (Appendix). Given the recent introduction of the second booster program in all adults in South Korea, further follow-up is needed to understand how protection changes in both persons at high risk and the general population.

The first limitation of our study is that our results might be affected by confounding bias if the first booster and second booster groups had different diagnostic intensity between groups. However, this difference in behavior that could have caused confounding would be smaller than in the general population, given that the study population included LTCF residents and immunocompromised persons, who receive the highest level of medical attention compared with other populations. Second, we were only able to estimate VE through days 30–53 of follow-up. Thus, the duration of protection against all infections will need to be monitored over a longer duration. Finally, during the peak surge of the Omicron outbreak in South Korea in February, persons who tested SARS-CoV-2–positive by self-administered rapid antigen tests might not have been included in the report. Despite these limitations, the second booster VE estimates against critical infection and death in persons at high risk were >50% compared with those in the first booster group, which suggests that additional doses continue to be an effective strategy to protect health in persons at higher risk.

In conclusion, our study provides real-world evidence that a second booster dose of mRNA COVID-19 vaccine provides substantially increased protection against critical infection and death in LTCF residents and immunocompromised persons receiving the booster dose. This protection will be key in the next wave of SARS-CoV-2 infection, when COVID-19 is again likely to pose a substantial burden to persons at high risk for serious health effects.

Appendix Additional information about effectiveness of second mRNA COVID-19 booster vaccine in immunocompromised persons and long-term care facility residents.
