# Supplementary material for: Effectiveness of Second mRNA COVID-19 Booster Vaccine in Immunocompromised Persons and Long-Term Care Facility Residents
Source: Emerg Infect Dis. 2022 Nov;28(11):2165–70. doi: 10.3201/eid2811.220918 (PMC9622254; doi:10.3201/eid2811.220918)
Supplement: Appendix — Additional information about effectiveness of second mRNA COVID-19 booster vaccine in immunocompromised persons and long-term care facility residents. [file 22-0918-Techapp-s1.pdf]

Article DOI: https://doi.org/10.3201/eid2811.220918

Effectiveness of Second mRNA COVID-19 Booster Vaccine in Immunocompromised Persons and Long-Term Care Facility Residents

Appendix

Appendix Table. Time-varying vaccine effectiveness against infection, critical infection, and death in persons who received the second booster vaccination compared with persons who received the first booster dose

	VE against all infection			VE against critical infection			VE against death		
Post-second booster	VE	UCI	LCI	VE	LCI	UCI	VE	LCI	UCI
0–14 d	29.51	28.59	30.42	51.61	44.02	58.17	53.99	46.11	60.72
15–30 d	32.77	31.65	33.87	53.85	44.17	61.85	52.08	41.38	60.83
>30 days	22.28	19.35	25.11	56.95	29.99	73.53	62.96	34.18	79.15

*LCI, lower confidence interval; UCI, upper confidence interval; VE, vaccine effectiveness.
